# Near-field probing of image phonon-polaritons in hexagonal boron nitride on gold crystals

**DOI:** 10.1126/sciadv.abn0627

**Published:** 2022-07-13

**Authors:** Sergey G. Menabde, Sergejs Boroviks, Jongtae Ahn, Jacob T. Heiden, Kenji Watanabe, Takashi Taniguchi, Tony Low, Do Kyung Hwang, N. Asger Mortensen, Min Seok Jang

**Affiliations:** ^1^ School of Electrical Engineering, Korea Advanced Institute of Science and Technology, Daejeon, Korea.; ^2^ Center for Nano Optics, University of Southern Denmark, Odense, Denmark.; ^3^ Nanophotonics and Metrology Laboratory, Swiss Federal Institute of Technology Lausanne (EPFL), Lausanne 1015, Switzerland.; ^4^ Center for Opto-Electronic Materials and Devices, Korea Institute of Science and Technology, Seoul, Korea.; ^5^ Research Center for Functional Materials, National Institute for Materials Science, Tsukuba, Ibaraki, Japan.; ^6^ International Center for Materials Nanoarchitectonics, National Institute for Materials Science, Tsukuba, Ibaraki, Japan.; ^7^ Department of Electrical and Computer Engineering, University of Minnesota, Minneapolis, MN, USA.; ^8^ Division of Nano and Information Technology, University of Science and Technology, Daejeon, Korea.; ^9^ Danish Institute for Advanced Study, University of Southern Denmark, Odense, Denmark.

## Abstract

Near-field mapping has been widely used to study hyperbolic phonon-polaritons in van der Waals crystals. However, an accurate measurement of the polaritonic loss remains challenging because of the inherent complexity of the near-field signal and the substrate-mediated loss. Here we demonstrate that large-area monocrystalline gold flakes, an atomically flat low-loss substrate for image polaritons, provide a platform for precise near-field measurement of the complex propagation constant of polaritons in van der Waals crystals. As a topical example, we measure propagation loss of the image phonon-polaritons in hexagonal boron nitride, revealing that their normalized propagation length exhibits a parabolic spectral dependency. Furthermore, we show that image phonon-polaritons exhibit up to a twice longer normalized propagation length, while being 2.4 times more compressed compared to the case of the dielectric substrate. We conclude that the monocrystalline gold flakes provide a unique nanophotonic platform for probing and exploitation of the image modes in low-dimensional materials.

## Teaser

Monocrystalline gold flakes provide a nanophotonic platform for low-loss image polaritons in van der Waals crystals.

## INTRODUCTION

Direct measurement of the polariton dispersion in low-dimensional van der Waals materials is possible via near-field mapping by the scattering-type scanning near-field optical microscope (s-SNOM) (*1*). In the s-SNOM experiments, the polaritons’ excitation and detection are typically performed by the same nanotip, and the recorded near-field interference pattern is due to the mode reflection at the material edge (*2*–*11*). Furthermore, in the case of the hyperbolic phonon-polaritons (HPPs) in hexagonal boron nitride (hBN), the near-field signal carries the contribution from the HPP waves launched by the s-SNOM excitation beam at the edge of an hBN slab (*3*–*6*, *12*).

Near-field probing of HPP can be simplified if polaritons are launched by metallic nanoparticles or metal edges with a larger scattering cross section (*4*, *7*, *13*, *14*). In this case, the near-field interference pattern is due to the superposition of the polariton field and the quasi-uniform excitation field of the s-SNOM (*4*). However, the small size and arbitrary shape of the gold nanoparticles still lead to a diverging wavefront of arbitrary shape (*4*, *14*, *15*). The combination of the nonplanar wavefront and the mixed near-field signal of different origins substantially complicates the near-field analysis, which often requires the development of prohibitively complex analytical models (*12*, *14*).

**Fig. 1.**
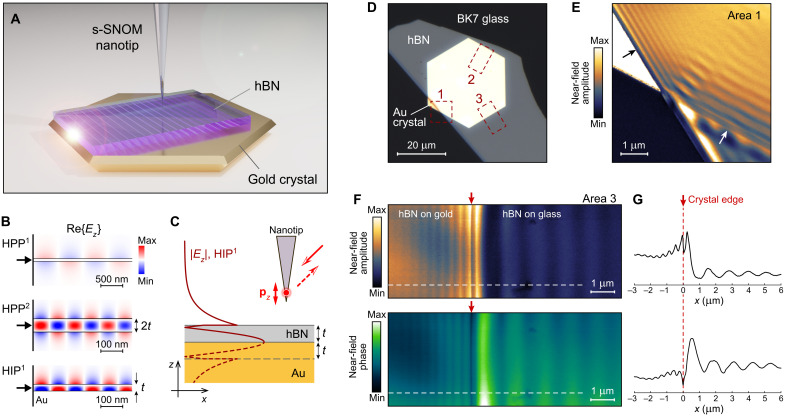
Near-field probing of image phonon-polaritons in hBN. (**A**) An atomically smooth monocrystalline gold flake provides a low-loss experimental platform for near-field probing of image polaritons. (**B**) Numerically calculated field profile of the first- and second-order HPP^1,2^ in a suspended hBN, and the first-order HIP^1^ in hBN on gold. Modes are launched at the port boundary (black arrow) and carry the same power; *t* = 30 nm. (**C**) The |*E*_z_| profile of the HIP^1^ eigenmode (solid) and its mirror image in gold (dashed), equivalent to the field profile of the second-order HPP^2^ mode in a twice thicker hBN. (**D**) Optical image of a 57-nm-thick hBN flake on a gold crystal; area 1 corresponds to the illustrative near-field image shown in (**E**), and areas 2 and 3 are where the HIP and HPP have been imaged, respectively. (E) Near-field map of a 63-nm-thick hBN slab cross-covering the edge of the gold crystal. The black arrow indicates the hBN edge on gold where HIP interference pattern is predominantly formed by the tip-launched modes; the white arrow indicates the gold edge under the hBN where HIP interference pattern is predominantly formed by the interference of HIP and the incident quasi-uniform excitation beam. (**F**) Maps of near-field amplitude (top) and phase (bottom) across area 3 marked in (D), where HIP and HPP are excited by the same gold edge under hBN. The red arrow indicates the position of the edge, which can be precisely determined by analyzing the interference fringes shown in (G). (**G**) Integrated profiles of the near-field interference fringes along the white dashed line shown in (F) for both near-field amplitude (top) and phase (bottom). The sharp dip in the phase profile indicates the position of the crystalline gold edge. All near-field images are obtained at the excitation frequency of 1480 cm^−1^.

A series of recent experimental works highlighted a new species of low-dimensional polaritons supported by the van der Waals crystals placed in proximity to a highly conductive metal—the image polaritons (*16*), resulting from the coupling of the collective charge oscillation in the polaritonic material with their images in the metal (*5*, *17*–*26*). Because of the lack of geometry-driven cutoff, image modes have been demonstrated to provide an unexcelled degree of field confinement into the nanometer-scale volumes (*22*–*26*). In this work, we use large-area gold crystals to rigorously study the dispersion of hyperbolic image phonon-polaritons (HIPs) in hBN slabs by near-field probing. The well-defined, ~20-μm-long crystalline gold edges efficiently launch HIP with a planar wavefront (Fig. 1A), significantly simplifying the near-field analysis. Furthermore, monocrystalline gold has an atomically flat surface with root mean square (RMS) roughness (*27*–*29*) as small as 1 Å (see fig. S1), which, along with the crystalline nature of hBN, practically eliminates roughness-mediated scattering of the propagating polaritons. Thus, our monocrystalline gold flakes can be used as an ultraflat substrate for highly compressed image polaritons in general, which are extremely sensitive even to atomic surface features (*30*).

By leveraging the physical properties of the gold crystals, we accurately measure the complex propagation constant of the HIP. In particular, the propagation loss (or the imaginary part of the propagation constant) of HIP is experimentally studied for the first time. Our experimental data agree well with the analytically calculated propagation loss in a system without scattering, showing a mean discrepancy of less than 4%. Furthermore, because of the practically scattering-free propagation, we detect a second-order image mode with a momentum of 5.9 × 10^5^ cm^−1^ even in a relatively lossy (naturally abundant) hBN crystal. So far, phonon-polaritons with such high momentum have been observed only in the low-loss isotopically enriched hBN (*6*). Last, our near-field investigation reveals that the fundamental image mode exhibits both stronger field confinement and longer normalized propagation length (in optical cycles) compared to the fundamental mode in the hBN on a dielectric substrate, notably similar to the behavior of image graphene plasmons (*17*). Thus, our findings highlight the unique dispersion property of image polaritons in general: The stronger field compression does not lead to a shorter polariton lifetime compared to the conventional two-dimensional (2D) modes in the same material.

## RESULTS

### Near-field mapping of phonon-polaritons

hBN is an anisotropic van der Waals crystal that supports propagating HPP in the two reststrahlen bands where the in-plane (ε*_xy_*) and out-of-plane (ε*_z_*) components of the permittivity tensor have an opposite sign of the real part (*3*, *31*). We restrict our study to the second reststrahlen band [1370 to 1610 cm^−1^; Re(ε*_xy_*) < 0, Re(ε*_z_*) > 0], which is accessible by the s-SNOM coupled with a quantum cascade laser.

Because of the hyperbolic dispersion, propagation of phonon-polaritons in hBN is restricted to a certain direction defined by the ratio ε*_xy_*/ε*_z_*, which leads to the quantization of the waveguide modes according to the linear scaling rule *k_z_t* ~ *l*, where *k_z_* is the out-of-plane component of the polariton wave vector, *t* is the hBN slab thickness, and *l* = 1,2,3… is the mode order (*31*, *32*). Figure 1B shows the field profile of the first- and the second-order HPP modes (HPP^1^ and HPP^2^, respectively), numerically calculated by the full-wave simulations. Because of the “reflection” by image charges, the fundamental HIP^1^ mode is equivalent to the symmetric HPP^2^ in a twice thicker hBN slab. Despite the tighter field confinement, the electric field of the HIP mode penetrates into the air above hBN at a distance ~ *t* and thus can couple to the s-SNOM nanotip acting as a *z*-oriented electric dipole (Fig. 1C). At the same time, the highly compressed field of HIP in a very thin hBN (*t* < 10 nm) may hinder their detection by s-SNOM (see discussion in the Supplementary Materials and fig. S2).

We obtain near-field images of an hBN flake on top of the hexagonal gold crystal on a borosilicate crown (BK7) glass substrate (Fig. 1D). Area 1 marks the sample structure where the illustrative near-field image of Fig. 1E has been obtained, and areas 2 and 3 show where the HIP and HPP dispersions have been measured, respectively.

In the absence of other scatterers, the HIP is efficiently launched by the s-SNOM tip and form a standing wave interference with the mode reflected by the hBN edge. At the same time, metallic particles efficiently launch HIP when placed underneath or on top of the hBN slab due to a much larger scattering cross section (*4*). Furthermore, since the slab is continuous in the latter case, the HIP launched by the tip does not experience strong reflection from the metal particles and mostly propagate away (see the Supplementary Materials); thus, the near-field interference pattern corresponds to the superposition of the HIP and the quasi-uniform illumination beam (*4*). The near-field image of an hBN slab crossing the crystalline gold edge (Fig. 1E) demonstrates the two cases of HIP excitation: The black arrow indicates the hBN edge on top of gold and the interference pattern of the standing wave with a period of λ_HIP_/2, where λ_HIP_ is the HIP wavelength. The white arrow indicates the crystalline gold edge underneath the hBN and the interference pattern formed by the HIP and the illumination beam with a period of λ_HIP_.

The edge of the gold crystal under the hBN launches polaritons in both directions—HIP on gold and HPP on a dielectric substrate (Fig. 1F). We note that HIP and HPP do not coexist on the same substrate since their dispersion does not match. By analyzing the interference fringes across the gold edge, it is possible to locate its position (Fig. 1G). In particular, the near-field phase map reveals the exact position of the edge due to the abrupt change of the complex near-field scattering coefficient.

### Extraction of complex propagation constant

Because polaritons are launched by the straight and long (≈ 20 μm ≫ λ_HIP_) crystalline gold edge and propagate in the structure with sub–1-nm surface roughness, the near-field interference pattern directly corresponds to the decaying field amplitude |*E_z_*| of the propagating modes with a planar wavefront. Therefore, the Fourier transform of the near-field interference fringes readily provides the value of the complex propagation constant, as explained below.

**Fig. 2.**
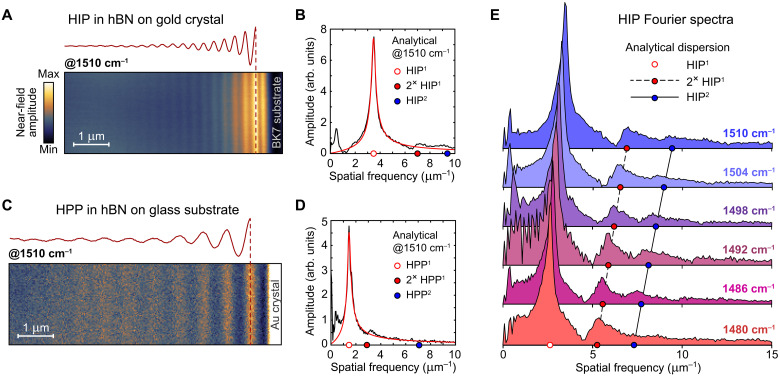
Fourier analysis of the near-field interference fringes. (**A**) Near-field image of the hBN slab on gold crystal near the crystal edge (area 2 in Fig. 1D), with clearly visible HIP fringes mapped at the excitation frequency of 1510 cm^−1^. The corresponding integrated fringes profile with removed background is shown above the near-field image. (**B**) Fourier spectrum (black) of the integrated fringes profile in (A) and its Lorentzian fit (red). The filled red circle indicates the double frequency of the HIP^1^ mode (empty red circle) where the signal from the tip-launched HIP^1^ is expected; the filled blue circle indicates the analytically predicted frequency of the second-order HIP^2^ mode. (**C** and **D**) The same as in (A) and (B) for the HPP launched by the gold edge and propagating in hBN on glass substrate (area 3 in Fig. 1D). (**E**) Fourier spectra of the HIP near-field interference fringes (shown in fig. S6) mapped at different frequencies in the middle of the second reststrahlen band. Fourier signal from the second-order HIP^2^ is clearly visible, in agreement with the analytical dispersion (filled blue circles). Strong double-frequency signal from the tip-launched HIP^1^ is also following its analytically predicted position (filled red circles).

**Fig. 3.**
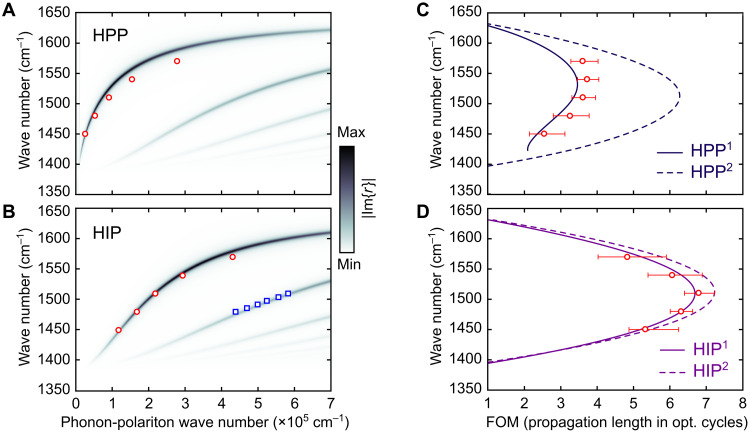
Dispersion and FOM of HPP and HIP modes. (**A**) Dispersion of the HPP modes calculated for a 57-nm-thick hBN slab on BK7 glass substrate (color map) and measured by near-field probing (red circles). (**B**) Calculated (color map) and measured dispersion of the HIP^1^ (red circles) and HIP^2^ (blue squares) in a 57-nm-thick hBN slab on a monocrystalline gold flake. (**C**) FOM of the first- and second-order HPP modes, calculated for the case in (A) (curves), and measured by near-field probing (red circles). (**D**) Calculated (curves) and measured by near-field probing (red circles) FOM of the fundamental HIP mode, whose dispersion is shown in (B) calculated (curves) and measured by near-field probing (red circles). Error bars correspond to 95% confidence interval of Lorentzian fitting; error bars for the momenta of HIP^1^ and HPP^1^ are smaller than the symbols and not shown.

Because of the planar wavefront of observed phonon-polaritons, it is possible to integrate fringe profiles across the large imaging area, which significantly improves the data quality. Figure 2A shows the near-field interference pattern and the integrated fringes profile (with removed background) of HIP in the hBN on gold (area 2 in Fig. 1D). Figure 2C shows similar data for HPP in the hBN on a glass substrate (area 3 in Fig. 1D). The corresponding Fourier spectra of the interference fringes (black) and their Lorentzian fit (red) are shown in the adjacent Fig. 2 (B and D) (see fig. S4 for the full spectral data and fig. S5 for the details on the background removing procedure). The peak position of the fitted Lorentzian gives the real part, and its full width at half maximum provides the imaginary part of the propagation constant. The filled red circles in the Fourier spectra indicate the double frequency of the HIP^1^ and HPP^1^ interference where the signal from the tip-launched standing wave is expected. A very weak double-frequency signal is present in all HIP spectra (fig. S4), but its small amplitude indicates that the fitted Lorentzian profile corresponds solely to the mode launched by the gold edge. Extracted wave numbers for the HPP^1^ and HIP^1^ modes are shown as red circles in Fig. 3 (A and B, respectively).

Furthermore, a weak Fourier signal is visible near the analytically predicted frequency of the ultraconfined HIP^2^ mode (filled blue circle in Fig. 2B); the HPP^2^ mode remains undetected in our experiments. We conducted a separate series of near-field measurements of HIP between 1480 and 1510 cm^−1^ (fig. S6) and obtained Fourier spectra of the interference fringes, as shown in Fig. 2E. Spectral data clearly show the HIP^2^ signal, which follows its analytically predicted dispersion (filled blue circles). Extracted wave numbers of the HIP^2^ mode are shown as blue squares in Fig. 3B, measured as local maxima of the Fourier spectra. The second-order image mode corresponds to the fourth-order HPP^4^ mode, which has been reported only once in the ultralow-loss isotopically enriched hBN slabs (*6*). Excitation of the ultraconfined HIP^2^ even in the relatively lossy (naturally abundant) hBN, as the one used in our experiments, highlights the negligible amount of scattering loss in the sample.

### Dispersion and loss analysis

As a figure of merit (FOM) of polariton damping, we use the normalized propagation length in optical cycles given by Re(*k_x_*)/[2πIm(*k_x_*)], where *k_x_* is the propagation constant. First, parameters of the hBN dielectric function are determined from the Raman spectroscopy of our samples and the previously reported data (*6*) (see the Supplementary Materials). Using the recovered hBN dielectric function, we analytically calculate the dispersion (color map in Fig. 3, A and B) and FOM (curves in Fig. 3, C and D) of the HPP and HIP modes. The FOM of the HPP^2^ mode exhibits a parabolic spectral dependency, maximizing at 1510 cm^−1^, where its propagation length is 1.65 times larger than that of the HPP^1^ mode (Fig. 3C). Naturally, a similar FOM is obtained for the HIP^1^ mode (Fig. 3D). However, the FOM of the HIP^2^ mode only has a slightly better maximal value. The drastic FOM difference between the HPP^1^ and HIP^1^ modes can be explained by a much smaller group velocity of the compressed HIP, while the polariton lifetime (material loss) practically does not change (fig. S12). The same dispersion property has been reported for the highly compressed image graphene plasmons (*17*, *18*). In other words, image polaritons, in general, have a highly compressed field, yet their lifetime is similar to that of the conventional 2D modes in the same material. Besides, because of the linear scaling of the HIP momentum with *t*, its FOM practically does not depend on the hBN thickness.

Experimentally obtained values of the FOM for the fundamental HIP^1^ mode are shown by red circles in Fig. 3D, in excellent agreement with the analytical predictions based on the recovered dielectric function of hBN, with a mean discrepancy of less than 4%. We note that our method provides an accurate value of loss only for the image modes since gold can be approximated as a perfect electric conductor at mid-infrared (mid-IR) frequencies. The possible presence of a few nanometer-thick air gaps between the gold and hBN does not affect the FOM (see the Supplementary Materials). However, given the agreement between the measured and calculated (without the gap) HIP momentum (Fig. 3B), we conclude that the thickness of the gap in the area of measurements is negligibly small.

Experimentally measured maximal FOM in our sample is 3.72 for the HPP (at 1540 cm^−1^) and 6.72 for the HIP (at 1510 cm^−1^), revealing a 1.8 times difference. When compared at the same excitation frequency of 1510 cm^−1^, the HIP FOM is ≈1.9 times larger than that of the HPP while having 2.4 times shorter wavelength (λ_HIP_ = 287 nm; λ_HPP_ = 689 nm).

The damping of phonon-polaritons in hBN strongly depends on its isotopic composition. Giles *et al.* (*6*) reported a greatly improved FOM of the HPP in the isotopically pure hBN, with the largest FOM ≈ 6.7 measured in the ^10^B 98.7% sample. Using the provided dielectric function for such isotopically pure hBN, we analytically predict an exceptionally large FOM = 19 for the HIP^1^ mode (calculated for the 100-nm-thick hBN at 1520 cm^−1^).

### Crystalline versus evaporated gold substrate

**Fig. 4.**
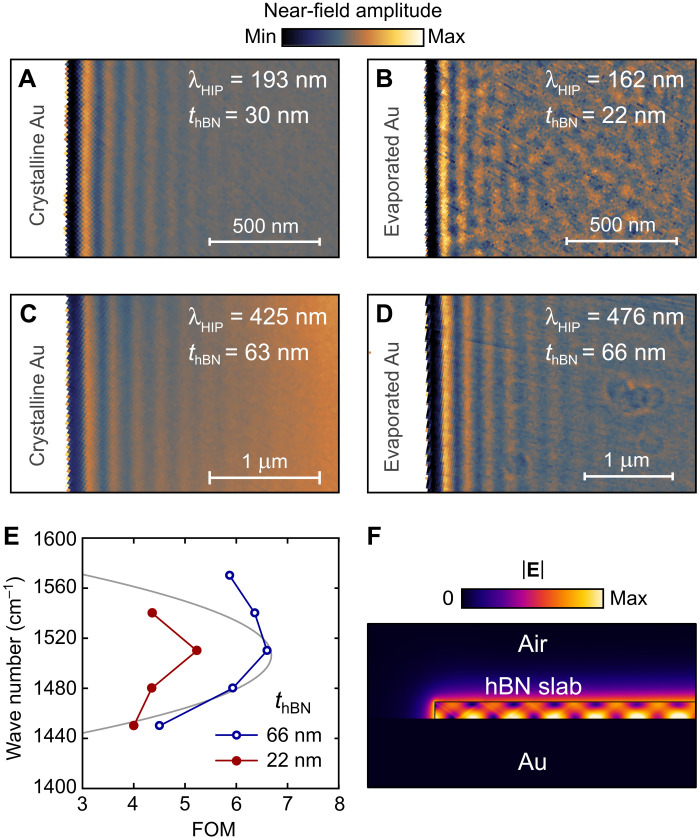
Roughness-mediated HIP scattering on evaporated gold. (**A** to **D**) Near-field scans of hBN slabs of different thicknesses (as noted) near the hBN edge: (A and C) on crystalline gold; (B and D) on evaporated gold. Excitation frequency is 1480 cm^−1^ in all cases. (**E**) Normalized propagation length of HIP in optical cycles, approximated from the near-field scans of hBN on evaporated gold: for *t* = 22 nm (red filled circles) and *t* = 66 nm (blue empty circles). The gray line shows the analytical solution for the HIP FOM. (**F**) Electric field amplitude profile of the HIP mode propagating to and reflecting from the hBN edge, numerically calculated by the full-wave simulations.

To estimate the impact of the gold roughness on HIP scattering loss, we have conducted the near-field probing of HIP on evaporated gold (RMS surface roughness ≈ 2 nm; fig. S1) in the hBN flakes of different thicknesses. In this case, only the standing wave interference fringes are available for analysis (fig. S9); therefore, we also obtain similar near-field interference images in 30- and 63-nm-thick hBN on gold crystals, as shown in Fig. 4 (A and C). The adjacent Fig. 4 (B and D) shows the near-field maps of 22- and 66-nm-thick hBN on evaporated gold. It is immediately evident that the roughness-mediated scattering on evaporated gold compromises the near-field probing of HIP in thinner hBN sample, while its effect is less pronounced in thicker hBN, where λ_HIP_ is three times larger. At the same time, negligible roughness of the crystalline gold does not contribute to HIP damping when *t* is reduced from 63 to 30 nm (Fig. 4, A and C).

We obtain the approximate FOM of the HIP on evaporated gold by analyzing the Fourier spectra of the interference fringes. In this case, an additional correction is required to compensate for the circular diverging wavefront and the double frequency of the fringes while assuming a perfect HIP reflection at the hBN edge (see the Supplementary Materials). Results of this approximate analysis are shown in Fig. 4E, correctly predicting the general tendency of the analytical FOM (gray curve) but only in the 66-nm-thick hBN (blue open circles), while HIPs in the 22-nm-thick slab propagate 20% less optical cycles at maximal FOM. Stronger HIP damping in thin hBN on evaporated gold can be understood from the HIP field amplitude distribution upon its reflection from the edge (Fig. 4F), where |*E*| is maximal immediately next to the gold surface. This, along with the shorter λ_HIP_, leads to a stronger HIP scattering by surface roughness.

## DISCUSSION

In conclusion, we demonstrate that the monocrystalline gold flakes provide a platform for precise near-field probing of image polaritons, where modes with planar wavefront are launched by the long crystalline gold edges and propagate on an ultraflat low-loss substrate. As a topical example, we measure the complex propagation constant of HIP in hBN within the second reststrahlen band. Our experimental data precisely follow the analytical prediction for the independently recovered hBN dielectric function. We demonstrate the larger field confinement and simultaneously longer normalized propagation length of the HIP modes compared to the HPP, notably similar to the behavior of the image graphene plasmons. This unique property potentially allows the combination of strong light-matter interaction and wave phenomena within a single nanophotonic platform.

## MATERIALS AND METHODS

### Sample preparation

Monocrystalline gold flakes were synthesized using the modified Brust-Schiffrin method (*33*) via thermolysis (*34*). An aqueous solution of the chloroauric acid (HAuCl_4_·3H_2_O in concentration 5 mM) was mixed with a solution of tetraoctylammonium bromide in toluene and stirred for 10 min at 5000 rpm. Then, the mixture was left to rest for approximately 10 min for the separation of aqueous and organic phases. The BK7 glass substrate was prepared by precleaning in an ultrasonic bath in acetone, isopropyl alcohol (IPA), and ultrapure water (Milli-Q). After blow-drying by nitrogen gas, the substrate was baked on a hot plate at 200°C for approximately 5 min for dehydration. Then, a few microliters of the organic phase was drop-casted onto a substrate, which was then left on the hot plate at 130°C for 24 hours. After that, the sample was cleaned in toluene at 75°C, acetone, and IPA, which removed most of the organic solvent. hBN nanoflakes were mechanically exfoliated from the bulk single crystal and transferred by the polydimethylsiloxane stamp on top of the gold crystals.

### Sample characterization

Near-field images were obtained by commercial s-SNOM (Neaspec GmbH), coupled with the tunable quantum cascade laser (Daylight Solutions, MIRcat). The Pt-coated atomic force microscope (AFM) tips (Nano World, ARROW-NCPt) were used with tapping frequency Ω around 260 kHz and oscillation amplitude 60 to 70 nm in a noncontact mode. The background-free interferometric signal (*35*) demodulated at third harmonic 3Ω was used to generate all near-field images. Samples were oriented in such a way that the plane of incidence of the s-SNOM illumination beam was never orthogonal to the gold edge, which significantly decreased the near-field background because of the strong scattering at the atomically sharp edge. The thickness of the hBN flakes was measured in the same AFM tapping mode as used for near-field imaging.

Raman spectroscopy of hBN on gold was performed using the LabRAM HR Evolution Visible-NIR system (HORIBA). The permittivity of the BK7 glass substrate at mid-IR frequencies was measured by ellipsometry (J.A. Woollam, IR-VASE).

## Supplementary Materials

### This PDF file includes:

Figs. S1 to S12 Table S1 References
