# Supplementary material for: Near-field probing of image phonon-polaritons in hexagonal boron nitride on gold crystals
Source: Sci Adv. 2022 Jul 13;8(28):eabn0627. doi: 10.1126/sciadv.abn0627 (PMC9278849; doi:10.1126/sciadv.abn0627)
Supplement: Supplementary file 1 — Figs. S1 to S12 Table S1 References [file sciadv.abn0627_sm.pdf]

Science Advances
AAAS

Supplementary Materials for

Near-field probing of image phonon-polaritons in hexagonal boron nitride on gold crystals

Sergey G. Menabde *et al.*

Corresponding author: N. Asger Mortensen, namo@mci.sdu.dk; Min Seok Jang, jang.minseok@kaist.ac.kr

Sci. Adv. **8**, eabn0627 (2022)
DOI: 10.1126/sciadv.abn0627

This PDF file includes:

Figs. S1 to S12
Tables S1
References

Supplementary Text

Near-field probing of HIP in very thin hBN

s-SNOM imaging of HPP in mono- and bi-layer hBN on SiO2 has been reported by S. Dai et al. (*36*). However, this study demonstrated only a single (rather a half of) near-field interference fringe at the edge of the hBN flake. Furthermore, HPP only at the lower end of the Reststrahlen band (at 1370–1385 cm^{-1}) was detected, because the mode confinement at higher frequencies was prohibitively large (effective mode index > 60). If such thin hBN is placed on gold, the HIP wavenumber at low frequencies would almost triple, leading to a much larger effective mode index of > 200 (λ_{HIP} < 40 nm), as theoretically predicted by Z. Yuan et al. (*20*). Therefore, SNOM detection of HIP would be extremely challenging, mostly limited by the in-plane spatial resolution of s-SNOM (~30 nm due to the nano-tip radius) and the small penetration depth of the evanescent field above the sample insufficient for out-coupling by the nano-tip. The calculated field penetration depth as a function of the hBN thickness (between 1 to 30 nm) and excitation frequency is shown in Fig. S2. This data shows that the image mode in hBN thinner than 10 nm would be difficult to detect in near-field, at least in the middle of the Reststrahlen band where FOM is maximal.

Dispersion of phonon-polaritons in hBN

In order to analytically calculate dispersion of phonon-polaritons in hBN slab, we employ a universal approach to find eigenmodes in a uniform three-layer structure with semi-infinite top and bottom layers as shown in Fig. S3. In this model, Medium 2 has properties of hBN, and Medium 1 can be either gold for the HIP case, or dielectric substrate for the HPP case. Assuming the TM-polarized mode propagating in the x-direction, the *ansatz* is made that the magnetic field in each medium m = 1,2,3 takes the form $\mathbf{H}^{(m)} = \hat{\mathbf{y}} H^{(m)}(z) e^{i[k_x x - \omega t]}$, where $k_x = 2\pi/\lambda_p$ is the polariton propagation constant, and λ_p is the polariton wavelength. Amplitude of the polaritons magnetic field $H^{(m)}$ is assumed to exponentially decay in z-direction and having constant complex amplitudes A_m and B_m:

$$H^{(1)}(z) = A_1 e^{ik_z^{(1)} z} + B_1 e^{-ik_z^{(1)} z}, z < 0;$$

$$H^{(2)}(z) = A_2 e^{ik_z^{(2)} (z-t)} + B_2 e^{-ik_z^{(2)} (z-t)}, 0 < z < t;$$

$$H^{(3)}(z) = A_3 e^{ik_z^{(3)} (z-t)} + B_3 e^{-ik_z^{(3)} (z-t)}, z > t.$$

In the above equations, $k_z^{(m)} = \sqrt{k_0^2 \varepsilon_m - k_x^2}$ is the z-component of the polariton wavevector in medium m, $k_0 = \omega/c$ is the free-space wavevector, and ε_m is the dielectric permittivity. Due to the anisotropy of hBN, the wavevector identity in Medium 2 takes the form $k_z^{(2)} = \sqrt{k_0^2 \varepsilon_{xy} - k_x^2 \frac{\varepsilon_{xy}}{\varepsilon_z}}$ where ε_{xy} and ε_z are the in-plane and out-of-plane hBN permittivity, respectively.

Substituting the equations for the magnetic field into the Maxwell's curl equation $\nabla \times \mathbf{H} - \varepsilon\varepsilon_0\, \partial\mathbf{E}/\partial t = \mathbf{J}$, we apply field continuity conditions for electric and magnetic fields at each interface: $E_x^{(m+1)} - E_x^{(m)} = 0$ and $H^{(m+1)} - H^{(m)} = 0$. Taking $A_1 = B_3 = 0$ (absence of reflected or incident waves in media "1" and "3"), we obtain a system of four linear equations for the four unknowns A_m and B_m. By searching for all possible solutions of the system of equations (if any), exact value of the complex propagation constant for all polariton eigenmodes supported by the system can be found. This powerful approach can be used to evaluate eigenmodes of multilayer structures with an arbitrary number of layers, or to calculate complex reflection and

transmission coefficients in the presence of an incident wave (e.g. when $B_3 = 1$), as employed to calculate the lossy HIP and HPP dispersion in Fig. 3 in the main text.

Type or paste text here. This should be additional explanatory text, such as: extended technical descriptions of results, full details of mathematical models, extended lists of acknowledgments, etc. It should not be additional discussion, analysis, interpretation, or critique.

Recovered hBN dielectric function from material data

Dielectric function of hBN is defined by its in-plane and out-of-plane optical phonon resonances, and its general TO-LO form for a given crystalline direction is given by:

$$\varepsilon(\omega) = \varepsilon_\infty \left(\frac{\omega_{LO}^2 - \omega^2 - i\Gamma\omega}{\omega_{TO}^2 - \omega^2 - i\Gamma\omega} \right),$$

where ω_{LO}, ω_{TO}, Γ and ε_∞ are the LO and TO phonon frequencies, phonon damping, and the high-frequency permittivity, respectively. hBN crystals with different proportion of boron isotopes ^{11}B and ^{10}B have slightly different phonon frequencies, but significantly different phonon lifetimes (*6*). Furthermore, frequency of the Raman active in-plane TO phonon mode linearly depends on the concentration ratio of the two isotopes (*6*). This makes it possible to learn the isotopic composition from the Raman spectrum. Raman spectra of three different hBN flakes used in our experiments are shown in Fig. S3, all having the sharp in-plane TO phonon peak at 1388 cm^{-1}, which gives the isotopic composition ^{10}B:$^{11}B \approx 80{:}20$.

According to the available experimental data from Ref. (*6*), provided for the full range of the isotopic ratio, we obtained parameters for the dielectric function of our samples, including 1 ps phonon lifetime which translates to $\Gamma = 5.3$ cm^{-1} for in-plane permittivity. Recovered parameters are given in the Table S1.

These parameters have been used to calculate analytical dispersion and FOM in Fig. 3 in the main text. Isotope ratio-related variation of loss in the out-of-plane dielectric function is negligible in the second reststrahlen band, therefore we adapt Γ as in the naturally abundant hBN.

Effect of air gap between gold and hBN

Since we transferred hBN flakes on top of the gold crystals, there is possibility that narrow air gap exists between the gold and hBN. Given the small thickness of our hBN (< 100 nm) and large lateral size of ~10 µm, waviness of hBN is limited and formation of large air gaps is unlikely. However, we calculated the dispersion of HIP mode in a four-layer structure with an air gap. As shown in Fig. S8, a few-nm air gap leads to a slightly smaller wavenumber, but does not affect the FOM. However, perfect agreement between the experimental and calculated dispersion in Fig. 3B of the main text suggests that thickness of the air gap is negligibly small in the area of the near-field measurements.

Reconstructing HIP dispersion from the near-field map of standing wave interference

As discussed in the main text, we measured near-field interference fringes in hBN on evaporated gold, where HIP are launched and scattered by the same s-SNOM nano-tip. In this case, circular polariton waves propagate from the tip towards the hBN edge, and get reflected from it, forming

a standing wave interference pattern (as shown in Fig. 4 of the main text). Figure S9 shows the full collection of near-field images of 22 and 66-nm thick hBN slabs on evaporated gold.

Interference fringes shown in Fig. S9 were integrated in a similar manner as those obtained at the crystalline gold. The amplitude of the near-field signal is proportional to the amplitude of the electric field, $s \propto |E_z|$, where E_z is the vertical component of the electric field under the tip. In order to compensate the energy dissipation in a diverging wavefront of polaritons, electric field amplitude is adjusted as $E_z \propto r^{-0.5}$, where r is the propagation distance from the excitation point. For simplicity, we assumed a 100% reflection of HIP at the hBN edge. Figure S10 shows the process of the fringes "reconstruction" by this approach for the near-field map on 66 nm-thick slab at 1480 cm^{-1}. First, integrated fringes (grey) were cleared of the background (red), so that their median is zero (black). We assume that the edge position coincides with the first bright maximum, so that remaining fringes can be adjusted for the distance $r/2$ between the tip and the edge. Reconstructed fringes are shown by green line. The Fourier spectrum of the reconstructed interference pattern is shown on the right panel, providing a good fit by the Lorentzian peak shape (red). When calculating the polariton wavenumber, the peak frequency was halved. In this way, we calculated the approximate FOM for HIP in hBN on evaporated gold shown in Fig. 4E in the main text.

<u>HIP and HPP reflection at crystalline gold edge</u>
Near-field interference fringes demonstrated in Fig. 3 and Fig. S4 show negligible contribution from the tip-launched modes that form a standing wave interference pattern with double spatial frequency. We suggest that, along with the more efficient polariton excitation by the gold edge, such purely edge-associated interference is due to a very weak reflection of the tip-launched modes at the gold edge when hBN slab is continuous. Numerical full-wave simulations provide the reflection coefficient $R < 0.3$ for the HPP, and $R < 0.2$ for the HIP near the frequency of the maximal FOM (Fig. S11b). Therefore, reflected diverging modes launched by the nano-tip form a very weak interference pattern, which is evident from the Fourier analysis of the interference fringes. The low reflection coefficient of the hyperbolic phonon-polaritons in hBN slab can be explained by the restricted propagation direction (*31*), so only a fraction of the scattered field couples to the backward-propagating polaritons. This is visible in the near-field amplitude profile of the reflected HPP and HIP modes (Fig. S11a) which propagate as a beam originating at the sharp gold edge, visualizing the only polariton wavevector direction available for the coupling to the scattered field. The similar picture is visible in the forward scattering direction.

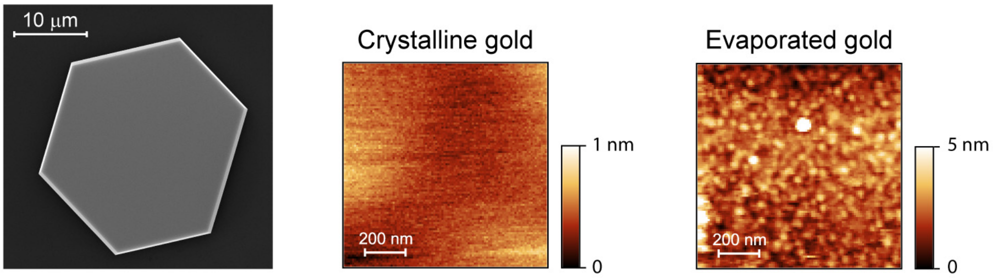

Fig. S1. Roughness of the monocrystalline gold flake and evaporated gold.
Left: SEM image of the hexagonal gold crystal. Center: AFM surface topography of the gold crystal with RMS roughness of ~0.2 nm. Right: AFM surface topography of ≈100 nm-thick evaporated gold deposited on Si/SiO_2 wafer with RMS roughness of ~2.3 nm.

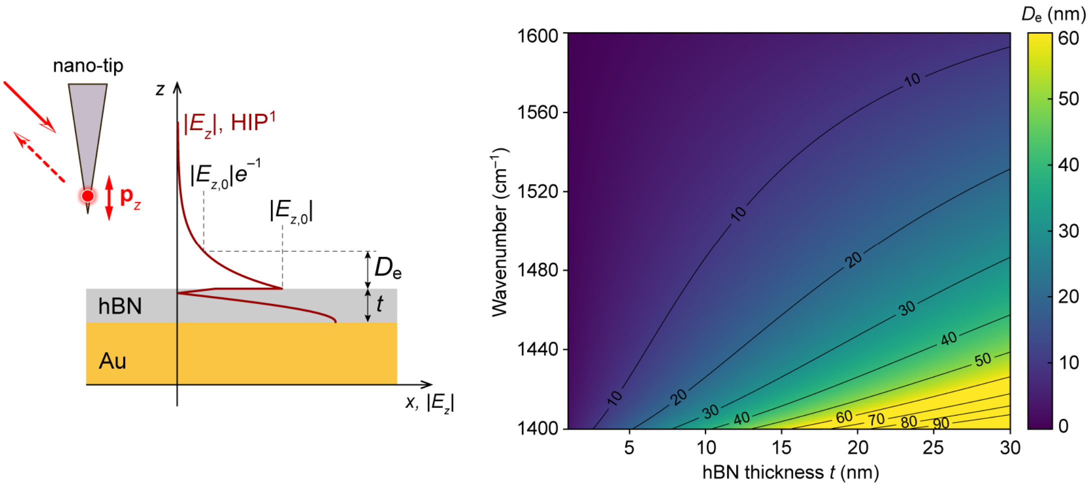

Fig. S2. Field penetration depth of the first-order HIP mode.

Left: definition of the penetration depth of the electric field E_z above the hBN. Right: map of its value calculated for the HIP^1 mode in the hBN of different thickness for frequencies within the second Reststrahlen band.

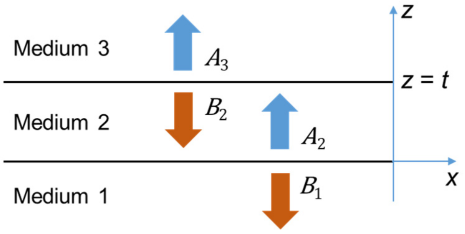

Fig. S3. Schematics of a three-layer structure used in analytical model.
Arrows indicate the direction of exponential decay of the polariton mode magnetic field $H(z)$ having complex amplitudes A_m and B_m.

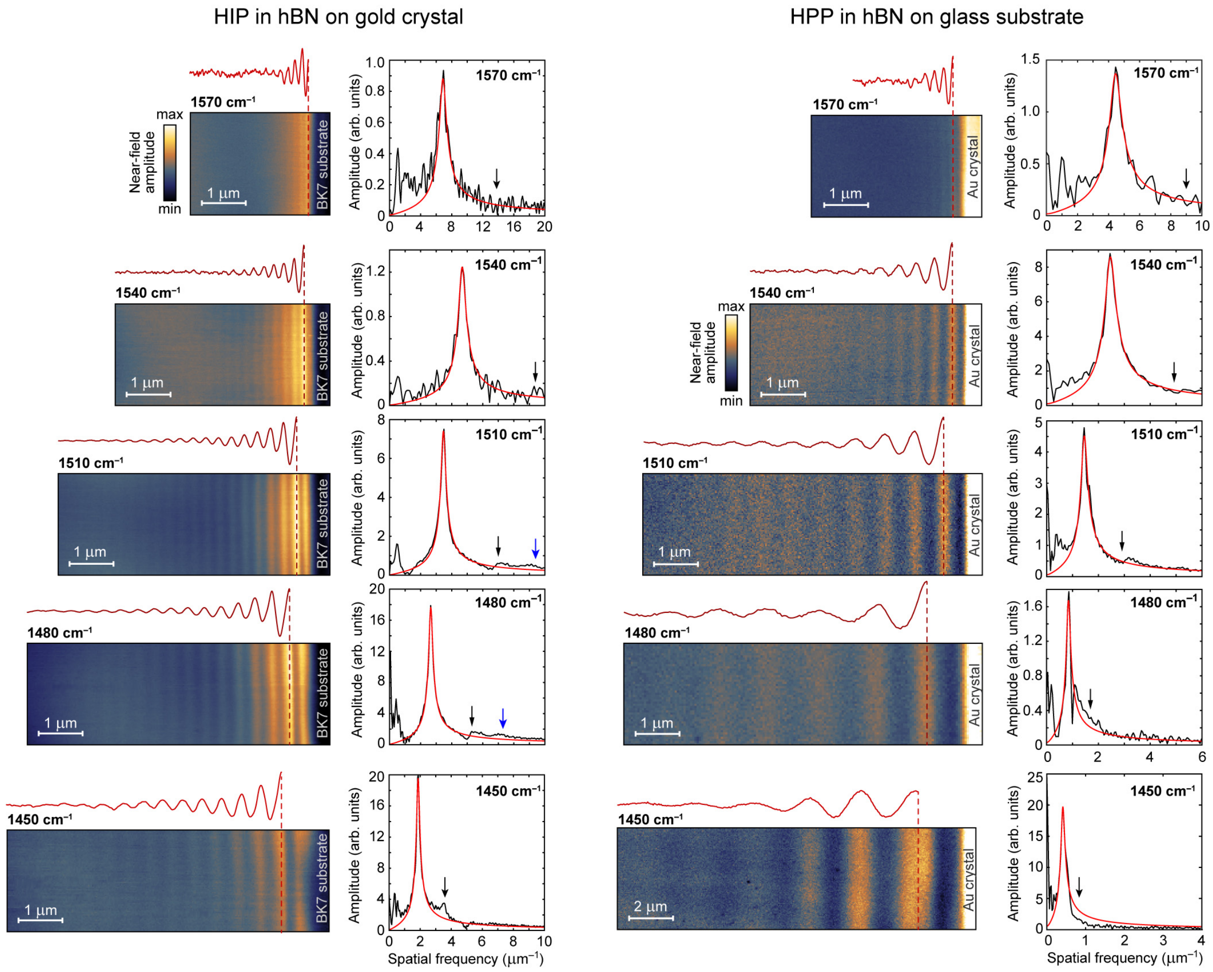

Fig. S4. Results of near-field probing of HIP and HPP and corresponding Fourier spectra. The black arrow in the Fourier spectra indicates the double-frequency signal from the tip-launched modes; the blue arrow indicates the analytically predicted frequency of the HIP^2 mode. HIP are measured in area "2" shown in Fig. 1D in the main text, and HPP are measured in area "3".

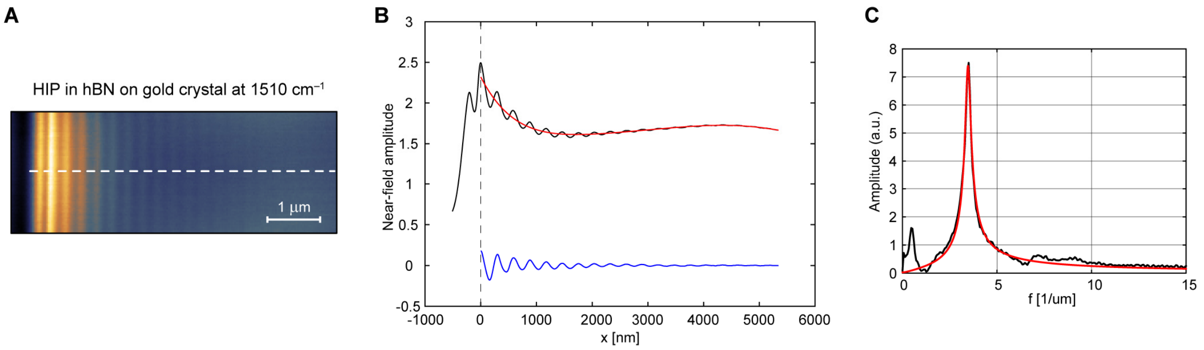

Fig. S5. Background removal procedure for near-field interference fringes.

(**A**) Near-field map of the HIP interference fringes in hBN on gold crystal measured at 1510 cm^{-1}. White dashed line shows the direction of fringes profile, which is integrated across the whole visible area. (**B**) As-measured profile of the near-field fringes (black), which is fitted with the sixth-order polynomial curve (red). The red curve (background signal) is subtracted from the measures fringes profile, resulting in the interference fringes with removed background (blue). (**C**) Fourier spectrum (black) of the “pure” interference fringes (blue curve in (B)) and its Lorentzian fit (red).

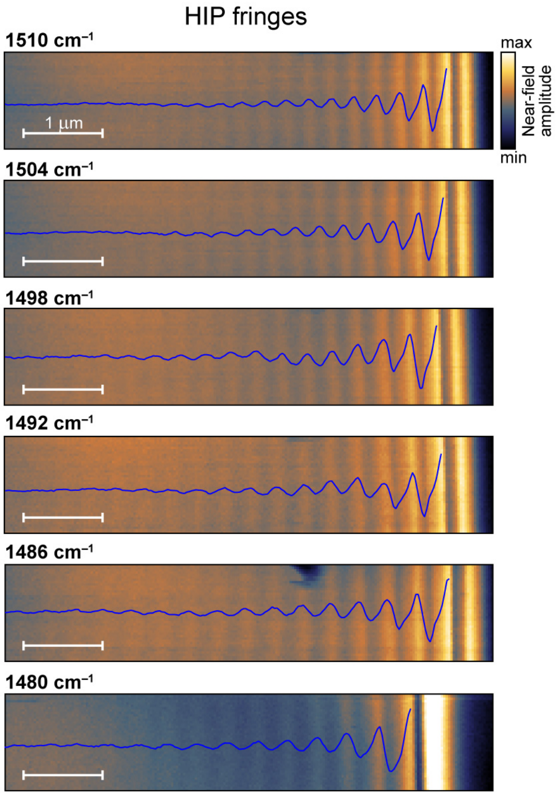

Fig. S6. Near-field mapping for the second-order HIP mode.

Near-field maps of the HIP interference fringes overlapped with their integrated profiles with removed background (blue curves) at several frequencies in the middle of the second Reststrahlen band; measured at the same area “3” Fig. 1D in the main text. All scale bars are 1 µm.

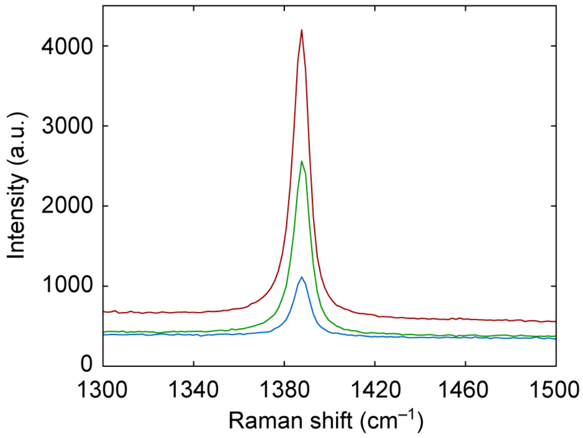

Fig. S7. Raman spectra of hBN.
Raman spectra of three hBN flakes of different thickness, all showing phonon peak at 1388 cm^{-1}.

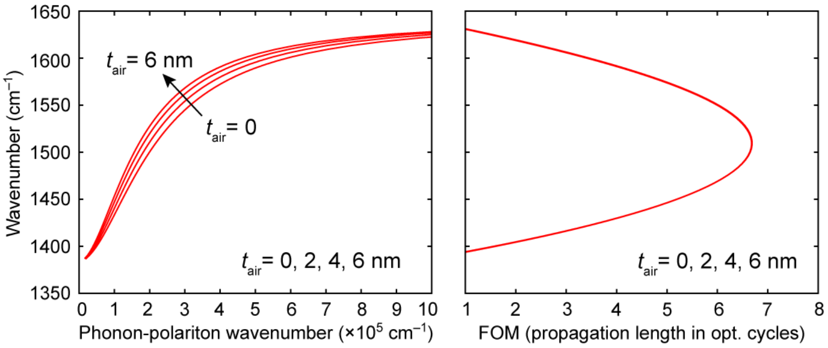

Fig. S8. Effect of the air gap thickness on HIP dispersion

Calculated HIP dispersion (left) and FOM (right) with air gap between gold and hBN of thickness t_{air}. Thickness of hBN corresponds to that of our sample t_{hBN} = 57 nm.

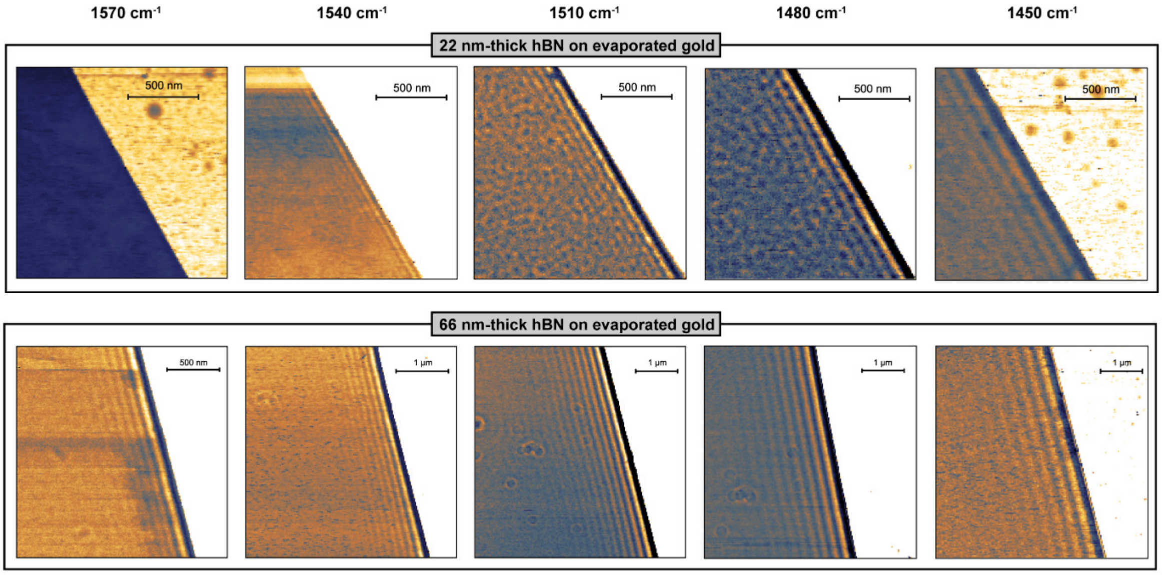

Fig. S9. Near-field probing of HIP on evaporated gold.

Near-field images of the hBN edge on evaporated gold for two samples of different thickness at the same frequencies.

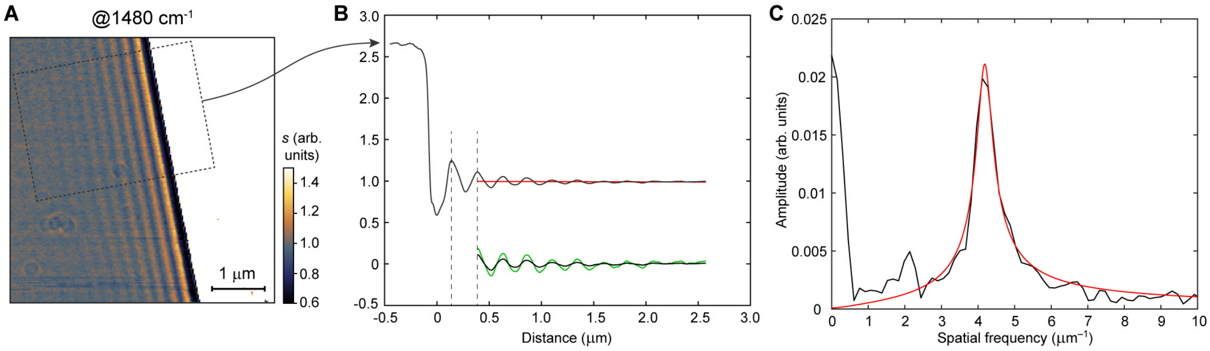

Fig. S10. Reconstruction of the interference fringes for the tip-launched HIP.
(**A**) Near-field image of hBN edge on evaporated gold, t = 66 nm. (**B**) Integrated interference fringes (grey), the background (red), and the result of removing the background (black). Edge position is assumed to be at the first bright maximum, therefore fringes are truncated at the second maximum to allow for the correct compensation. Reconstructed fringes (green) are analyzed by the Fourier transform as shown in (**C**): Fourier spectrum of the reconstructed interference fringes (black) and its Lorentzian fit (red).

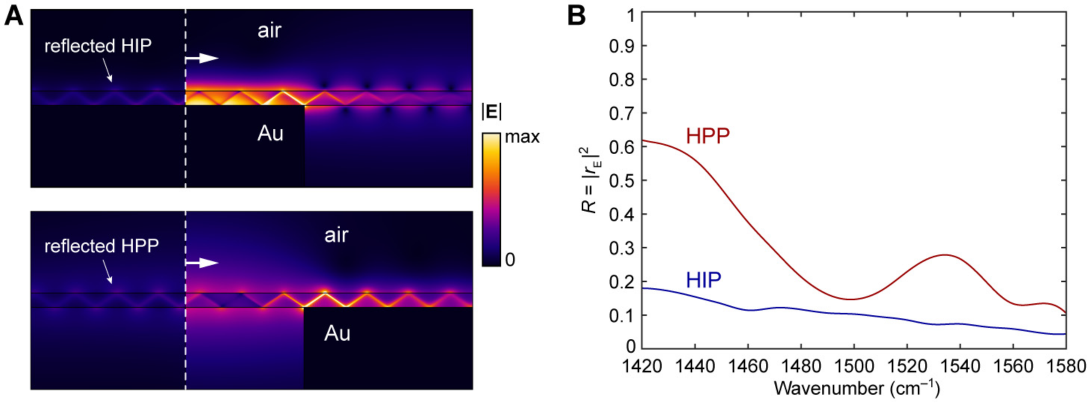

Fig. S11. Numerical analysis of polaritons reflection at the gold edge.

(**A**) Electric field amplitude profile calculated by the full-wave FEM simulations for HIP (top) and HPP (bottom) modes launched at the numerical port boundary condition (white dashed line with arrow; excitation frequency is 1510 cm^{-1}) and propagating towards the gold edge. hBN slab of thickness 60 nm is considered to be in contact with gold and suspended in air when no gold substrate is present. (**B**) Reflection coefficient $R = |r_E|^2$ for HPP and HIP modes, where the complex field reflection coefficient r_E is obtained from the full-wave simulations at different frequencies.

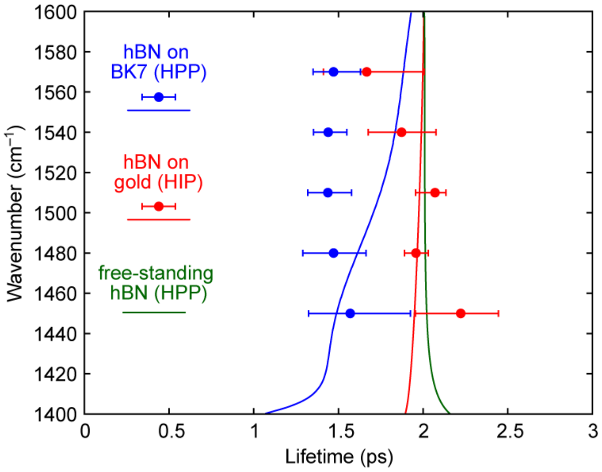

Fig. S12. Polaritons lifetime analysis.

Analytically calculated (curves) lifetimes of HIP in hBN on gold (red), HPP in hBN on BK7 glass substrate (blue), and HPP in free-standing hBN (green); hBN thickness 57 nm. The lifetime is calculated as $\tau = L_{\mathrm{p}}/v_{\mathrm{g}}$, where $L_{\mathrm{p}} = 1/\mathrm{Im}\{k_x\}$ is the propagation length, $v_{\mathrm{g}} = \partial\omega/\partial k_{\mathrm{HIP}}$ is the group velocity, and k_{HIP} is the polariton momentum. For the experimental values (data points with error bars), group velocity is calculated analytically, and the propagation length is obtained from the near-field images. Error bars correspond to the 95% confidence interval for the Lorentzian fitting to the Fourier spectra. Note that the analytically predicted lifetimes of the HIP and the HPP in free-standing hBN are very similar, while the experimental HIP lifetime is close to the theoretical limit, indicating the negligible external loss from scattering and absorption in the substrate.

	ω_{LO}, cm^{-1}	ω_{TO}, cm^{-1}	Γ, cm^{-1}	ε_{∞}
In-plane	1641.7	1387.6	5.3	5.1
Out-of-plane	838.8	779.0	3	2.95

Table S1. Parameters of the hBN dielectric function.
Extracted parameters of the hBN dielectric function used for analytical and numerical modeling.

REFERENCES AND NOTES

1. X. Chen, D. Hu, R. Mescall, G. You, D. N. Basov, Q. Dai, M. Liu, Modern scattering-type scanning near-field optical microscopy for advanced material research. *Adv. Mater.* **31**, 1804774 (2019).

2. S. Dai, Q. Ma, T. Andersen, A. S. Mcleod, Z. Fei, M. K. Liu, M. Wagner, K. Watanabe, T. Taniguchi, M. Thiemens, F. Keilmann, P. Jarillo-Herrero, M. M. Fogler, D. N. Basov, Subdiffractional focusing and guiding of polaritonic rays in a natural hyperbolic material. *Nat. Commun.* **6**, 6963 (2015).

3. S. Dai, Z. Fei, Q. Ma, A. S. Rodin, M. Wagner, A. S. McLeod, M. K. Liu, W. Gannett, W. Regan, K. Watanabe, T. Taniguchi, M. Thiemens, G. Dominguez, A. H. C. Neto, A. Zettl, F. Keilmann, P. Jarillo-Herrero, M. M. Fogler, D. N. Basov, Tunable phonon polaritons in atomically thin van der Waals crystals of boron nitride. *Science* **343**, 1125–1129 (2014).

4. S. Dai, Q. Ma, Y. Yang, J. Rosenfeld, M. D. Goldflam, A. McLeod, Z. Sun, T. I. Andersen, Z. Fei, M. Liu, Y. Shoa, K. Watanabe, T. Taniguchi, M. Thiemens, F. Keilmann, P. Jarillo-Herrero, M. M. Fogler, D. N. Basov, Efficiency of launching highly confined polaritons by infrared light incident on a hyperbolic material. *Nano Lett.* **17**, 5285–5290 (2017).

5. A. Ambrosio, M. Tamagnone, K. Chaudhary, L. A. Jauregui, P. Kim, W. L. Wilson, F. Capasso, Selective excitation and imaging of ultraslow phonon polaritons in thin hexagonal boron nitride crystals. *Light-Sci. Appl.* **7**, 27 (2018).

6. A. J. Giles, S. Dai, I. Vurgaftman, T. Man, S. Liu, L. Lindsay, C. T. Ellis, N. Assefa, I. Chatzakis, T. L. Reinecke, J. G. Tischler, M. M. Fogler, J. H. Edgar, D. N. Basov, J. D. Caldwell, Ultralow-loss polaritons in isotopically pure boron nitride. *Nat. Mater.* **17**, 134–139 (2018).

7. G. X. Ni, A. S. McLeod, Z. Sun, L. Wang, L. Xiong, K. W. Post, S. S. Sunku, B. Y. Jiang, J. Hone, C. R. Dean, M. M. Fogler, D. N. Basov, Fundamental limits to graphene plasmonics. *Nature* **557**, 530–533 (2018).

8. A. Y. Nikitin, P. Alonso-González, S. Vélez, S. Mastel, A. Centeno, A. Pesquera, A. Zurutuza, F. Casanova, L. E. Hueso, F. H. L. Koppens, R. Hillenbrand, Real-space mapping of tailored sheet and edge plasmons in graphene nanoresonators. *Nat. Photon.* **10**, 239–243 (2016).

9. A. Bylinkin, M. Schnell, M. Autore, F. Calavalle, P. Li, J. Taboada-Gutierrez, S. Liu, J. H. Edgar, F. Casanova, L. E. Hueso, P. Alonso-Gonzalez, A. Y. Nikitin, R. Hillenbrand, Real-space observation of vibrational strong coupling between propagating phonon polaritons and organic molecules. *Nat. Photon.* **15**, 197–202 (2021).

10. G. Álvarez-Pérez, T. G. Foland, I. Errea, J. Taboada-Gutierrez, J. Duan, J. Martin-Sanchez, A. I. F. Tresguerres-Mata, J. R. Matson, A. Bylinkin, M. He, W. Ma, Q. Bao, J. I. Martin, J. D. Caldwell, A. Y. Nikitin, P. Alonso-Gonzalez, Infrared permittivity of the biaxial van der Waals semiconductor α-MoO_3 from near- and far-field correlative studies. *Adv. Mater.* **32**, 1908176 (2020).

11. W. Ma, P. Alonso-González, S. Li, A. Y. Nikitin, J. Yuan, J. Martín-Sánchez, J. Taboada-Gutierrez, I. Amenabar, P. Li, S. Vélez, C. Tollan, Z. Dai, Y. Zhang, S. Sriram, K. Kalantar-Zadeh, S.-T. Lee, R. Hillenbrand, Q. Bao, In-plane anisotropic and ultra-low-loss polaritons in a natural van der Waals crystal. *Nature* **562**, 557–562 (2018).

12. B. Liao, X. Guo, D. Hu, F. Zhai, H. Hu, K. Chen, C. Luo, M. Liu, X. Yang, Q. Dai, A multibeam interference model for analyzing complex near-field images of polaritons in 2D van der Waals microstructures. *Adv. Func. Mater.* **29**, 1904662 (2019).

13. E. Yoxall, M. Schnell, A. Y. Nikitin, O. Txoperena, A. Woessner, M. B. Lundeberg, F. Casanova, L. E. Hueso, F. H. L. Koppens, R. Hillenbrand, Direct observation of ultraslow hyperbolic polariton propagation with negative phase velocity. *Nat. Photon.* **9**, 674–678 (2015).

14. G. Ni, A. S. McLeod, Z. Sun, J. R. Matson, C. F. B. Lo, D. A. Rhodes, F. L. Ruta, S. L. Moore, R. A. Vitalone, R. Cusco, L. Artús, L. Xiong, C. R. Dean, J. C. Hone, A. J. Millis, M. M. Fogler, J. H. Edgar, J. D. Caldwell, D. N. Basov, Long-lived phonon polaritons in hyperbolic materials. *Nano Lett.* **21**, 5767–5773 (2021).

15. J. Duan, R. Chen, J. Li, K. Jin, Z. Sun, J. Chen, Launching phonon polaritons by natural boron nitride wrinkles with modifiable dispersion by dielectric environments. *Adv. Mater.* **29**, 1702494 (2017).

16. S. G. Menabde, J. T. Heiden, J. D. Cox, N. A. Mortensen, M. S. Jang, Image polaritons in van der Waals crystals. *Nanophotonics* **11**, 2433–2452 (2022).

17. S. G. Menabde, I. H. Lee, S. Lee, H. Ha, J. T. Heiden, D. Yoo, T. T. Kim, T. Low, Y. H. Lee, S. H. Oh, M. S. Jang, Real-space imaging of acoustic plasmons in large-area graphene grown by chemical vapor deposition. *Nat. Commun.* **12**, 938 (2021).

18. K. V. Voronin, U. A. Aguirreche, R. Hillenbrand, V. S. Volkov, P. Alonso-Gonzalez, A. Y. Nildtin, Nanofocusing of acoustic graphene plasmon polaritons for enhancing mid-infrared molecular fingerprints. *Nanophoton.* **9**, 2089–2095 (2020).

19. A. R. Echarri, J. D. Cox, F. J. G. de Abajo, Quantum effects in the acoustic plasmons of atomically thin heterostructures. *Optica* **6**, 630–641 (2019).

20. Z. Yuan, R. Chen, P. Li, A. Y. Nikitin, R. Hillenbrand, X. Zhang, Extremely confined acoustic phonon polaritons in monolayer-hBN/metal heterostructures for strong light-matter interactions. *ACS Photonics* **7**, 2610–2617 (2020).

21. A. Fali, S. T. White, T. G. Folland, M. He, N. A. Aghamiri, S. Liu, J. H. Edgar, J. D. Caldwell, R. F. Haglund, Y. Abate, Refractive index-based control of hyperbolic phonon-polariton propagation. *Nano Lett.* **19**, 7725–7734 (2019).

22. M. B. Lundeberg, Y. Gao, R. Asgari, C. Tan, B. Van Duppen, M. Autore, P. Alonso-Gonzalez, A. Woessner, K. Watanabe, T. Taniguchi, R. Hillenbrand, J. Hone, M. Polini, F. H. L. Koppens, Tuning quantum nonlocal effects in graphene plasmonics. *Science* **357**, 187–191 (2017).

23. D. A. Iranzo, S. Nanot, E. J. C. Dias, I. Epstein, C. Peng, D. K. Efetov, M. B. Lundeberg, R. Parret, J. Osmond, J.-Y. Hong, J. Kong, D. R. Englund, N. M. R. Peres, F. H. L. Koppens, Probing the ultimate plasmon confinement limits with a van der Waals heterostructure. *Science* **360**, 291–295 (2018).

24. I. Epstein, D. Alcaraz, Z. Huang, V.-V. Pusapati, J.-P. Hugonin, A. Kumar, X. M. Deputy, T. Khodkov, T. G. Rappoport, J.-Y. Hong, N. M. R. Peres, J. Kong, D. R. Smith, F. H. L. Koppens, Far-field excitation of single graphene plasmon cavities with ultracompressed mode volumes. *Science* **368**, 1219–1223 (2020).

25. I. H. Lee, D. Yoo, P. Avouris, T. Low, S. H. Oh, Graphene acoustic plasmon resonator for ultrasensitive infrared spectroscopy. *Nat. Nanotechnol.* **14**, 313–319 (2019).

26. I.-H. Lee, M. He, X. Zhang, Y. Luo, S. Liu, J. H. Edgar, K. Wang, P. Avouris, T. Low, J. D. Caldwell, S.-H. Oh, Image polaritons in boron nitride for extreme polariton confinement with low losses. *Nat. Commun.* **11**, 3649 (2020).

27. S. Boroviks, C. Wolff, J. Linnet, Y. Yang, F. Todisco, A. S. Roberts, S. I. Bozhevolnyi, B. Hecht, N. A. Mortensen, Interference in edge-scattering from monocrystalline gold flakes. *Opt. Mater. Express* **8**, 3688–3697 (2018).

28. K. J. Kaltenecker, E. Krauss, L. Casses, M. Geisler, B. Hecht, N. A. Mortensen, P. U. Jepsen, N. Stenger, Mono-crystalline gold platelets: A high-quality platform for surface plasmon polaritons. *Nanophoton.* **9**, 509–522 (2020).

29. J.-S. Huang, V. Callegari, P. Geisler, C. Bruning, J. Kern, J. C. Prangsma, X. Wu, T. Feichtner, J. Ziegler, P. Weinmann, M. Kamp, A. Forchel, P. Biagioni, U. Sennhauser, B. Hecht, Atomically flat single-crystalline gold nanostructures for plasmonic nanocircuitry. *Nat. Commun.* **1**, 150 (2010).

30. N. Zhang, W. Luo, L. Wang, J. Fan, W. Wu, M. Ren, X. Zhang, W. Cai, J. Xu, Strong in-plane scattering of acoustic graphene plasmons by surface atomic steps. *Nat. Commun.* **13**, 983 (2022).

31. J. D. Caldwell, A. V. Kretinin, Y. Chen, V. Giannini, M. M. Fogler, Y. Francescato, C. T. Ellis, J. G. Tischler, C. R. Woods, A. J. Giles, M. Hong, K. Watanabe, T. Taniguchi, S. A. Maier, K. S. Novoselov, Sub-diffractional volume-confined polaritons in the natural hyperbolic material hexagonal boron nitride. *Nat. Commun.* **5**, 5221 (2014).

32. G. Alvarez-Perez, K. V. Voronin, V. S. Volkov, P. Alonso-Gonzalez, A. Y. Nikitin, Analytical approximations for the dispersion of electromagnetic modes in slabs of biaxial crystals. *Phys. Rev. B* **100**, 235408 (2019).

33. M. Brust, M. Walker, D. Bethell, D. J. Schiffrin, R. Whyman, Synthesis of thiol-derivatised gold nanoparticles in a two-phase liquid-liquid system. *J. Chem. Soc. Chem. Commun.* **7**, 801–802 (1994).

34. B. Radha, G. U. Kulkarni, A real time microscopy study of the growth of giant au microplates. *Cryst. Growth Des.* **11**, 320–327 (2011).

35. N. Ocelic, A. Huber, R. Hillenbrand, Pseudoheterodyne detection for background-free near-field spectroscopy. *Appl. Phys. Lett.* **89**, 101124 (2006).

36. S. Dai, W. Fang, N. Rivera, Y. Stehle, B.-Y. Jiang, J. Shen, R. Y. Tay, C. Ciccarino, Q. Ma, D. Rodan-Legrain, P. Jarillo-Herrero, E. H. T. Teo, M. M. Fogler, P. Narang, J. Kong, D. N. Basov, Phonon polaritons in monolayers of hexagonal boron nitride. *Adv. Mater.* **31**, 1806603 (2019).
